# Homeostatic Roles of the Proteostasis Network in Dendrites

**DOI:** 10.3389/fncel.2020.00264

**Published:** 2020-08-14

**Authors:** Erin N. Lottes, Daniel N. Cox

**Affiliations:** Neuroscience Institute, Georgia State University, Atlanta, GA, United States

**Keywords:** dendrite, proteostasis network, ribosome, chaperone, autophagy, ubiquitin-proteasome system (UPS), developmental homeostasis, neurological disease

## Abstract

Cellular protein homeostasis, or proteostasis, is indispensable to the survival and function of all cells. Distinct from other cell types, neurons are long-lived, exhibiting architecturally complex and diverse multipolar projection morphologies that can span great distances. These properties present unique demands on proteostatic machinery to dynamically regulate the neuronal proteome in both space and time. Proteostasis is regulated by a distributed network of cellular processes, the proteostasis network (PN), which ensures precise control of protein synthesis, native conformational folding and maintenance, and protein turnover and degradation, collectively safeguarding proteome integrity both under homeostatic conditions and in the contexts of cellular stress, aging, and disease. Dendrites are equipped with distributed cellular machinery for protein synthesis and turnover, including dendritically trafficked ribosomes, chaperones, and autophagosomes. The PN can be subdivided into an adaptive network of three major functional pathways that synergistically govern protein quality control through the action of (1) protein synthesis machinery; (2) maintenance mechanisms including molecular chaperones involved in protein folding; and (3) degradative pathways (*e.g.*, Ubiquitin-Proteasome System (UPS), endolysosomal pathway, and autophagy. Perturbations in any of the three arms of proteostasis can have dramatic effects on neurons, especially on their dendrites, which require tightly controlled homeostasis for proper development and maintenance. Moreover, the critical importance of the PN as a cell surveillance system against protein dyshomeostasis has been highlighted by extensive work demonstrating that the aggregation and/or failure to clear aggregated proteins figures centrally in many neurological disorders. While these studies demonstrate the relevance of derangements in proteostasis to human neurological disease, here we mainly review recent literature on homeostatic developmental roles the PN machinery plays in the establishment, maintenance, and plasticity of stable and dynamic dendritic arbors. Beyond basic housekeeping functions, we consider roles of PN machinery in protein quality control mechanisms linked to dendritic plasticity (*e.g.*, dendritic spine remodeling during LTP); cell-type specificity; dendritic morphogenesis; and dendritic pruning.

## Introduction

Some of Ramon y Cajal’s most famous drawings are of dendrites, and though much of our fascination in his work is due to Cajal’s skill in rendering each branch in minute detail, some of the appeal is naturally due to the sheer variety in shape and size of cells. In an illustration of a single slide, he might capture three or four different types of cells, crowding around each other in the same tiny slice of tissue (Garcia-Lopez et al., 2010). Cajal described the sight as “the nerve cell, the highest caste of organic elements, [appears] with its giant arms stretched out, like the tentacles of an octopus, to the provinces on the frontiers of the external world, to watch for the constant ambushes of physico-chemical forces” (qtd. in Ramón y Cajal, 1989). Our fascination with complicated dendritic arbors is not misplaced, for the shape of neurons evinces their function: the ornate arbors of hippocampal neurons need to integrate numerous inputs, and the bipolar cells of the retina only require two processes to facilitate rapid neural communication. The unique cell-specific structures of neuronal processes are vital to the function of each cell and to the function of the brain in its entirety. The brain relies on precise and reliable relationships between cells, and the cells, in turn, rely on their specific dendritic arbors to maintain proper connections between themselves and the larger cellular community.

The cell body alone is a whirring hub of activity, and the axon can stretch for incredible distances, making synaptic connections at many points along its length. Each neuron can participate in thousands of synaptic connections, which total over 100 trillion synapses in a human neocortex alone (Hanus and Schuman, 2013; Tang et al., 2001). The spatial architecture of a dendritic arbor is key to ensuring its appropriate synaptic inputs, and, thus, its proper function. There are three major physical requirements for dendrites to function correctly: (1) the arbor must cover its receptive field; (2) the branch pattern must be suited to the type and amount of incoming signals; and (3) the dendrites must be plastic, changing with both development and experience (Jan and Jan, 2010).

**FIGURE 1:**
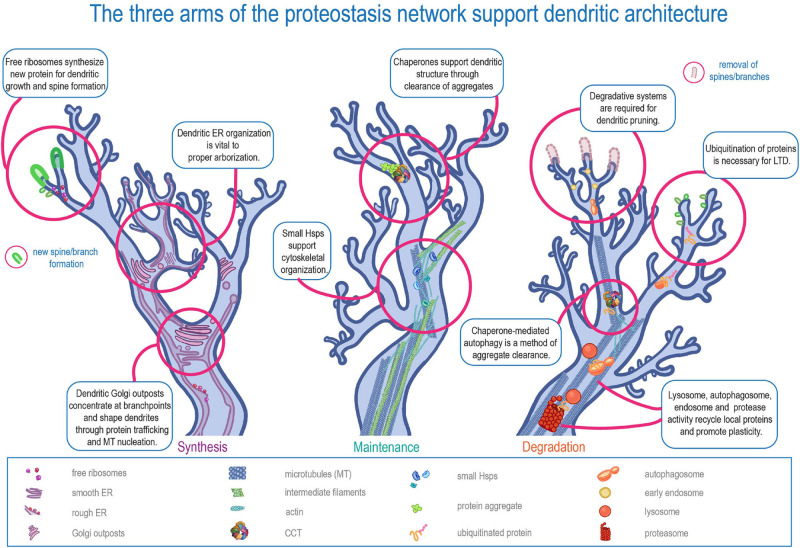
The three arms of the proteostasis network in supporting dendritic architecture. Schematic representation of the three major arms of proteostasis (Synthesis, Maintenance, and Degradation) in regulating distinct aspects of dendritic development and function.

In short, dendrites must be both stable and dynamic. The half-life of a synaptic protein is a few days at most, but the main branches of the arbor may need to be maintained for the course of an organism’s life (Cohen et al., 2013). Another conflict: cytoskeletal proteins are moved via “slow” transport, which can be less than eight millimeters a day, but the physical changes in shape and size of dendritic spines can begin less than an hour after induction of long term potentiation (LTP) (Ostroff et al., 2018) – and protein level fluctuation starts even earlier (Bosch et al., 2014; Maday and Holzbaur, 2014; Ostroff et al., 2018; Hafner et al., 2019). Dendrites require protein transport from the cell body, but these examples illustrate that dendrites cannot simply rely on transport to maintain proteostasis. Instead, dendritic protein quality control systems must be in place to meet the needs of stability and plasticity. These systems include free ribosomes and dendritic endoplasmic reticulum (ER) tubules that facilitate local translation, cytoplasmic chaperones that monitor protein maintenance in dendrites, and dendritic autophagosomes, endosomes, lysosomes, and proteasomes that control localized protein recycling and turnover (Figure 1).

Protein quality control is a sub-component of proteostasis involving protein synthesis, maintenance, and degradation (Klaips et al., 2018). This review will address the three arms of protein quality control in dendrites, from a protein’s ribosomal “birth” through its maintenance or “maturation” by chaperones, and eventually to its autophagic and ubiquitin-mediated “death.” Each proteostatic arm, when disrupted, is associated with a variety of neurological disorders, highlighting the importance of these proteostatic components to neurons, especially. Many studies have been conducted on protein quality control in cell-stress conditions (Chaari, 2019; Muranova et al., 2019; Hetz and Kaufman, 2020; Yerbury et al., 2020); however, this review will mainly address the function of each arm in homeostatic conditions. Furthermore, many of the studies discussed in this review use genetic manipulation to dissect the role of PN genes and their protein products in homeostasis. Here we discuss accumulated evidence of dendritic expression and localization of proteins and organelles that point to compartment-specific roles of PN machinery in regulating dendritic development and plasticity. With that said, an important technical caveat of the molecular genetic manipulations is that while PN genes can be genetically disrupted in a cell-type specific manner in some organisms, these manipulations are not necessarily targeted to a specific compartment and instead effect the entire cell – axon, dendrite and soma. As such, it can be technically challenging to fully disentangle putative contributions of the somatic PN from the dendritic PN. Nevertheless, that PN machinery is differentially trafficked onto dendrites, and supports biological processes such as local translation, indicates that at least somatodendritic PN machinery mechanistically functions in protein quality control linked to dendritic morphogenesis, cell-type specificity, and plasticity.

### Protein Synthesis

The first arm of the protein quality control system, protein synthesis, controls translation of mRNA into protein. Ribosomes, located both in the rough ER and freely in the cytosol, shepherd the transition of mRNA to protein (Ainsley et al., 2014; Depaoli et al., 2018). Often organized in complexes comprised of many individual ribosomal subunits (Genuth and Barna, 2018), ribosomes interact with a variety of other proteins, including Ribosomal Associated Proteins (RAPs), kinases, and phosphatases, which facilitate the production of all proteins in the cell (Heise et al., 2014; Genuth and Barna, 2018).

It has long been known that free, or cytosolic, ribosomes are present in dendrites (Tiedge and Brosius, 1996; Hanus and Schuman, 2013; Genuth and Barna, 2018). In fact, ribosomal proteins have often been tagged with fluorescent markers in order to visualize dendritic arbors (Hill et al., 2012). Selectively disrupting ribosomal function has been found to affect many aspects of axonal and dendritic morphology (Perry and Fainzilber, 2014; Slomnicki et al., 2016; Genuth and Barna, 2018). This is not surprising: both axons and dendrites extend for great distances, forming intricate, complicated connections – and these polarized structures must be maintained for much longer than the lives of other cell types, requiring continual protein synthesis.

### Protein Synthesis and Trafficking in Development and Maintenance of Arbors

Due to the special proteostatic demands of dendrites, it is logical that both cytosolic and ER protein translation would occur on-site. Solely depending on vesicular trafficking of essential proteins would be slow and energetically costly (Hanus and Schuman, 2013; Perry and Fainzilber, 2014). In rat brain slices, it was found that in a five-minute period – absent of any exogenous stimulation – approximately 60% of observed dendritic spines underwent active translation (Hafner et al., 2019).

The levels of some ribosomal subunits have dramatic effects on neurite formation, and some ribosomal transcription factors – transcription factors that regulate the expression of ribosomal subunits – have been specifically examined for their pro-neuritic function (Gomes et al., 2011; Das et al., 2017; Hetman and Slomnicki, 2019; Baral et al., 2020). Ribosomal subunits have been found to be required for correct dendritic arbor formation, as the knockdown of *RpS3* and *RpL22*, 40S and 60S ribosomal subunit proteins, respectively, shrinks and simplifies arbors in Class IV (CIV) nociceptive dendritic arborization sensory neurons in *Drosophila melanogaster* (Olesnicky et al., 2014; Lin et al., 2015). Knockdown of many other ribosomal subunits have been found to alter CIV dendritic morphology in *Drosophila* larvae, such as *RpL7, RpL36A, RpS2, RpS13*, and *RpS17* (Das et al., 2017; Nanda et al., 2018; Table 1). Mutations of *RpL7* and *RpL36A* also resulted in reductions in dendritic F-actin and microtubule levels as well as redistribution of F-actin towards the soma, which may contribute to the observed gross morphological defects (Das et al., 2017).

**TABLE 1:** *Protein Synthesis Dendritic Phenotypes* proteins involved in regulating protein synthesis cause a variety of dendritic phenotypes when manipulated.

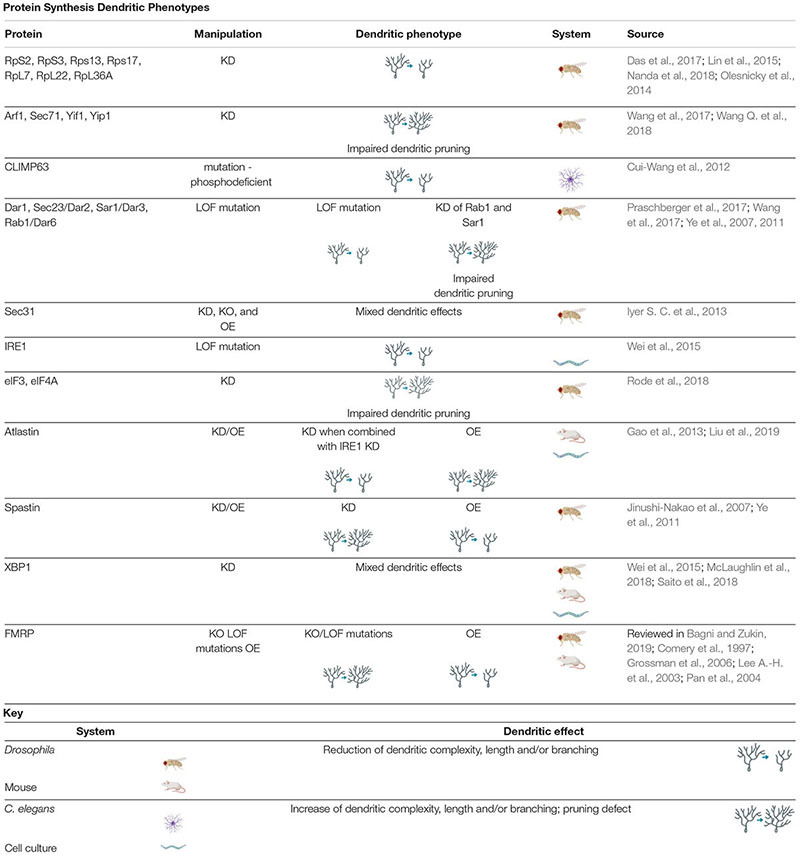

Though some studies indicate that dendritic translation is more heavily dependent on free ribosomes than the rough ER (Koltun et al., 2020), it has been found that a healthy ER is necessary for correct dendritic arbor formation (Cui-Wang et al., 2012). Neurons have evolved a unique spatial organization of the secretory system, with satellite ER and Golgi outposts found outside of the soma (Horton and Ehlers, 2003; Aridor et al., 2004; Ye et al., 2007; Iyer S. C. et al., 2013). This specialized neuronal secretory system is necessary for neuronal polarization and the asymmetric growth and branching that distinguishes axons from dendrites (Horton and Ehlers, 2003; Horton et al., 2005; Ye et al., 2007). The ER runs in a “tubular” form in axons and the straightaways of dendrites but creates more complicated satellite networks at dendritic branch points (Liu et al., 2019). Proper ER formation may underlie correct dendritic arbor formation (Cui-Wang et al., 2012). For example, CLIMP63, an integral ER membrane protein, guides the elongation of ER tubules to the distal ends of dendritic processes when “activated” by phosphorylation. When an “inactivated” phosphodeficient version of CLIMP63 was introduced into rat hippocampal neurons, the neurons produced fewer proximal branches. Conversely, when a phosphomimetic version of CLIMP63 was introduced, there was an increase in branch number (Cui-Wang et al., 2012). These results are similar to those of experiments manipulating the protein Atlastin – an ER tubule-binding protein which, when mutated is a cause of Hereditary Spastic Paraplegia, discussed in sections “Protein Synthesis Linked to Neurological Disease” and “Protein Maintenance in Disease” (Fink, 2013; Ozdowski et al., 2015). In *Drosophila*, knockdown of *atlastin* orthologs leads to ER network fragmentation in dendrites, though dendritic arborization defects only resulted when the knockdown was combined with a knockdown of *inositol-requiring enzyme-1* (*IRE1)* (see section “Protein Synthesis Linked to Neurological Disease”) (Liu et al., 2019; Summerville et al., 2016). Furthermore, overexpression of Atlastin in mouse cortical neurons led to increased dendritic growth both *in vivo* and *in vitro* (Gao et al., 2013).

Other components of the secretory pathway, such as Golgi outposts – specialized Golgi compartments in dendrites – have also been proven to be crucial for dendritic growth (Ye et al., 2007; Zhou et al., 2014). Dendritic Golgi outposts, which are distinct from the Golgi apparatus in the soma, depend on a RhoA-Rho kinase (Rock) signaling pathway for formation and deployment into dendrites (Quassollo et al., 2015). Satellite Golgi outposts supply the plasma membrane needed to support growth in distal dendrites, transported via vesicle trafficking. The COPII machinery needed to facilitate dendritic vesicle trafficking is also implicated in dendritic growth and branching. Mutations in COPII components such as the coat proteins *Sec13*, *Sec23*, *Sec24*, and *Sec31*, as well as GTPases *Rab1* and *Sar1* cause reductions in dendritic growth and branching in *Drosophila* CIV neurons (Ye et al., 2007; Iyer S. C. et al., 2013). Interestingly, genes involved in the secretory pathway have also been found to be necessary for the developmentally timed dendritic pruning, a regressive process, that occurs in *Drosophila* pupae during metamorphosis. Defects in the function of *Arf1, Sec71, Yif1* and *Yip1*, as well as *Rab1* and *Sar1*, have all been found to severely disrupt dendritic pruning via dysregulation of the ER-to-Golgi network (Wang et al., 2017; Wang Q. et al., 2018). It has also been suggested that Golgi outposts may play a role in supporting local translation and protein trafficking (Steward and Schuman, 2003; Ye et al., 2007). Finally, Golgi outposts have also been found to act as microtubule-organizing centers in dendrites, a role which is essential for the formation and maintenance of the dendritic arbor (Ori-McKenney et al., 2012; Yang and Wildonger, 2020). For excellent, recent reviews on the role of the secretory pathway in neurons and the role of the Golgi complex in neurological disorders see Kennedy and Hanus (2019) and Caracci et al. (2019).

### Protein Synthesis and Cell-Type Specificity

Mutations of some secretory pathway proteins – (*e.g.*, Sec23, Sar1 and Rab1) have been found to cause developmental dendritic defects (Table 1), yet mutations in similar secretory proteins (*e.g.*, Sec23A, Sec23B, Sec24D, and Sar1b) have been linked to non-neuronal effects in humans (Ye et al., 2007; Praschberger et al., 2017). Instead of impacting neuronal morphology, the latter group of secretory proteins are found to disrupt bone formation and cause lipid absorption disorders and anemia when mutated (Praschberger et al., 2017). These findings indicate that there may be tissue-specific dependence on different components of the synthesis and secretory systems, tailored to the unique needs of each tissue. In development, certain ribosome biogenesis factors – which are crucial to ribosomal complex assembly – are specifically expressed in stem cells, and cellular ribosomal content is thought to change as select ribosomes are recruited depending on cell fate (Gabut et al., 2020).

Ribosomal distribution and cell reliance are more than just tissue-specific. The differences in ribosomal reliance are even brain-region specific. It has been discovered that *Drosophila* larval neuroblasts show differential responses to CRISPR-mediated knockdown of ribosome biogenesis factors. Neuroblasts of the mushroom body proliferated much longer after loss of two ribosome biogenesis factors than did other neuroblast types (Baral et al., 2020). Recent discoveries have also revealed that ribosomal protein paralogues eRpL22 and eRpL22-like show cell-type specific patterning in the *Drosophila* eye in addition to developmental-dependent fluctuations in their expression (Gershman et al., 2020).

The cell-type specific patterning of protein synthesis machinery may also contribute to the diversity of dendritic morphologies. As in the *Drosophila* eye, sensory neurons in the *Drosophila* larval body wall show differing expression and dependence on ribosomal expression (Iyer E. P. R. et al., 2013; Das et al., 2017). As alluded to above, a *Sec31* loss-of-function mutation caused decreased dendritic length and branching in *Drosophila* CIV sensory neurons, however, there was no effect of *Sec31* mutation in classes of sensory neurons with simpler dendritic arbors. Furthermore, Sec31 overexpression also resulted in decreased dendritic growth and branching in complex CIV neurons, whereas Sec31 overexpression in the morphologically simpler Class I (CI) sensory neurons resulted in enhanced dendritic growth and branching, revealing cell-type specific differences in dendritic development and homeostasis (Iyer S. C. et al., 2013). This phenomenon has also been observed in *C. elegans*, where knockdown of *IRE1*, which encodes a protein monitoring ER content (discussed later in section “Protein Synthesis Linked to Neurological Disease”), causes severe reductions in dendritic branching in neurons with complex dendritic arbors, but not those with simpler arbors (Wei et al., 2015). In *Drosophila*, ribosomal genes were found to be more highly enriched in the dendritically complex CIV neurons relative to the dendritically simpler CI neurons (Iyer E. P. R. et al., 2013). This could indicate that more complex neurons require higher levels of protein synthesis in general, or, alternatively, are more sensitive to perturbations in protein synthesis and secretory systems. It could also be the case that specific ribosomal proteins are required for complex neurons because those neurons depend heavily on “specialized ribosomes” (discussed in section “Protein Synthesis and Plasticity”) to translate the select subset of proteins required to develop such complex arbors.

### Protein Synthesis and Plasticity

The formation of the ER and presence of free ribosomes is necessary for the development and stability of the dendritic arbor in many types of neurons (Martínez et al., 2018), but ribosomes also play an important role in dendritic plasticity. Free ribosomes in the cytoplasm allow compartments like the dendrites and axon to independently respond to experiences, and ribosomes themselves may have more discretion than previously imagined. Instead of identical machines that non-discriminately transcribe any strand of mRNA that comes their way, ribosomes may selectively transcribe mRNA depending on cellular conditions or post-translational modifications (Genuth and Barna, 2018; Ferretti and Karbstein, 2019).

The term “specialized ribosomes”, also referred to as the “ribosome filter hypothesis” is currently used to refer to the idea that ribosomes, able to translate many different strands of mRNA, may have selective control over the prioritization of competing strands (Mauro and Edelman, 2007; Shi et al., 2017; Genuth and Barna, 2018). This selective control springs from the ribosomal subunit composition and/or stoichiometry of the ribosomal complex itself – selectivity may arise through slight architectural distinctions between subunit isoforms or paralogs, or dynamically through changes in cell conditions like temperature or post-translational modifications like acetylation (Gerst, 2018; Guo, 2018; Li and Wang, 2020). Alternatively, ribosomal subunits and their paralogs may be tuned to translate specific sets of proteins, and the translation of these proteins may be up- or down-regulated based on the production and degradation of the ribosomal subunits themselves (Komili et al., 2007; Ferretti and Karbstein, 2019).

Ribosome specialization is an issue complicated by the sheer variety of proteins involved in translation. RAPs (Ribosomal Associated Proteins), kinases, and phosphatases are all a part of the “ribo-interactome” (Albert et al., 2019; Genuth and Barna, 2018; Heise et al., 2014). mRNA translated by ribosomes is capped at the 5′ end, and the cap requires recruitment of eukaryotic initiation factors (eIFs) such as eIF4E and eIF4G before they, in turn, recruit the ribosome (Ostroff et al., 2017; Choi et al., 2018; Das Sharma et al., 2019). Some eIFs are even preferentially involved in different stages of dendritic growth and maintenance, such as eIF4A and eIF3, which have been found to be required for dendritic pruning in *Drosophila* pupae (Rode et al., 2018). Additionally, some ribosomes can directly bind with mRNA using Internal Ribosome Entry Sites (IRES); however, even while skipping the “middle man”, they still depend on a whole host of other proteins that assist with initiation, elongation, and termination of the mRNA (Sutton and Schuman, 2005; Genuth and Barna, 2018).

Specialized ribosomes may be necessary for the differences in protein expression between cell types, and thus for the formation of unique dendritic arbors (Kennedy and Hanus, 2019). Interestingly, differences in the rate of mRNA translation have even been uncovered between proximal and distal dendritic branches of the same arbor (Ouwenga et al., 2017; Wu et al., 2016; Xue and Barna, 2012), implicating ribosomal specialization in the distinction and dynamics of cell compartments.

It is well known that dendritic spines physically change in response to activity as part of LTP, and ribosomal activity is thought to be an integral part of that change (Chidambaram et al., 2019; Chirillo et al., 2019; Harris, 2020). Ribosomal mRNAs have been found to be enriched in dendrites (Ohashi and Shiina, 2020), and dendritic levels of mRNA fluctuate with synaptic activity, especially those of immediate early genes such as Activity-regulated cytoskeletal associated protein (Arc) (Sutton and Schuman, 2005; Jakkamsetti et al., 2013; Na et al., 2016; Ostroff et al., 2017; Choi et al., 2018). Using a specially designed fluorescent reporter, Arc was visualized in real time in dendrites as it was translated, appearing only fifteen seconds after synaptic stimulation with glutamate (Na et al., 2016), too quickly for traditional transport mechanisms. Surprisingly, it appeared in the dendrites but not the spines themselves, lending credence to the idea that synapses might share resources (Hanus and Schuman, 2013). Local translation may occur at the dendrite level, with proteins transported short distances to activated synapses: this is aligned with the synaptic tagging model, in which synapses share pools of resources, and allocated proteins are somehow “tagged” in order to recruit them to specific synapses (Frey and Morris, 1997; Ainsley et al., 2014; Rogerson et al., 2014). Supporting this theory, ribosome numbers have been found to be elevated in dendritic shafts following LTP induction during the persistent phase of LTP, which is dependent on protein synthesis, and mRNA translation is up-regulated in both the dendrites and soma following LTP induction (Ostroff et al., 2018; Chirillo et al., 2019; Koltun et al., 2020).

### Protein Synthesis Linked to Neurological Disease

Dyshomeostasis of protein synthesis can be lethal. Ribosomopathies, caused by loss-of-function mutations of ribosomes or ribosome biogenesis factors, clearly exemplify the importance of individual ribosome subunits in organismal health. Ribosomopathies, such as Diamond-Blackfan anemia, often cause congenital birth defects and heightened cancer risk (Shi et al., 2017). For reviews of ribosomopathies, see Farley-Barnes et al. (2019) and Kampen et al. (2020).

Though ribosomopathies have consequences throughout the body, some ribosomal subunit mutations are connected specifically to neurological disorders, such as those linked to cases of Autism Spectrum Disorder (ASD) and intellectual disability (ID) (Hetman and Slomnicki, 2019; Choe and Cho, 2020). Defects in ribosome function have also been connected to neurodegenerative diseases such as Alzheimer’s disease (AD) (Ding et al., 2005; Hernández-Ortega et al., 2016) and Parkinson’s disease (PD) (Taymans et al., 2015), and ribosomal frameshifting is implicated in repeat expansion disorders such as Huntington’s disease (HD) and Amyotrophic Lateral Sclerosis (ALS) (Gao et al., 2017).

Disruption of ER organization can also underlie neurological disease, though this may be due to the loss of its organizational organelle contacts rather than its peripheral protein synthesis functions (Fowler et al., 2019). Mutations in the protein Atlastin – an ER-tubule binding protein previously discussed – are responsible for 10% of autosomal dominant cases of Hereditary Spastic Paraplegia, a disease characterized by progressive weakness and loss of motor control in the lower limbs (Fink, 2013; Ozdowski et al., 2015). Changes in ER organization are characteristic of several mutations underlying forms of Hereditary Spastic Paraplegia, including changes to *atlastin, spastin, reticulon 2*, *REEP1* and *2*, and *protrudin*, among others (Blackstone, 2018; Fowler et al., 2019). For a recent review of the role of the ER in axons and neurodegeneration, see Öztürk et al. (2020). The neurological disease is thought to stem mainly from axon degeneration, and disruptions in axonal regeneration and bouton number have been reported with mutations in Hereditary Spastic Paraplegia-associated genes (Rao et al., 2016; Summerville et al., 2016). However, loss-of-function experiments with *atlastin* and other molecules have also been found to severely disrupt gross dendritic morphology and dendritic spine formation (Fink, 2013; Liu et al., 2019; Shih and Hsueh, 2018). Moreover, mutations in *spastin*, which encodes a microtubule-severing AAA ATPase, are known to be the most frequent cause of autosomal dominant spastic paraplegia, and have also been shown to lead to reductions in dendritic arbor complexity (Jinushi-Nakao et al., 2007; Ye et al., 2011). There is accumulating evidence that disruption of the dendritic arbor may also contribute to this disease, and may be an interesting angle at which to study Hereditary Spastic Paraplegia in the future.

The ER does not need to be physically malformed to contribute to neurological disease, as ER stress has been linked to several neurodegenerative conditions (Hetz and Mollereau, 2014; Plate and Wiseman, 2017; Martínez et al., 2018; McLaughlin et al., 2018; Morris et al., 2018). ER stress occurs when the amount of unfolded proteins in the ER reaches an unmanageable level, triggering the unfolded protein response (UPR) (Martínez et al., 2018). Many proteins in the UPR pathway have been linked to neurodegenerative diseases. For example, x-box protein 1 (XBP1) has been found to be neuroprotective in *Drosophila* expressing amyloid-β42 in neurons (Marcora et al., 2017). XBP1 is a downstream effector of IRE1, one of the triggers that initiates part of the UPR cascade upon sensing unfolded protein buildup in the ER (Wei et al., 2015). Interestingly, IRE1, which can initiate both cytoprotective and apoptotic cascades (Sano and Reed, 2013), has also been found in *C. elegans* to be required for proper dendritic arborization – implicating the UPR in not only neurodegeneration but neurodevelopment as well (Wei et al., 2015). This is supported by a recent finding that showed activation of XBP1 through IRE1 may promote developmental dendritic outgrowth through the transcriptional activation of BDNF (Saito et al., 2018). For a recent review on the molecular details of the UPR and its role in disease, see (Hetz and Kaufman, 2020).

Fragile X syndrome (FXS) is a clear example of how a neurological disorder could arise from dysregulation of protein synthesis. FXS results from a mutation in the *fmr1* gene and is the leading inherited cause of ASD (Greenblatt and Spradling, 2018). The FXS mutation and subsequent silencing of the *fmr1* gene prevents the production of FMRP (Fragile X Mental Retardation Protein), which normally regulates initiation of translation (Das Sharma et al., 2019; Liu et al., 2018). FMRP creates a complex with initiation proteins, including the aforementioned eIF4E, and then binds ribosomes to control translation of nascent protein (Das Sharma et al., 2019). Without FMRP, Arc becomes constitutively expressed, and overall translation is disinhibited (Park et al., 2008; Na et al., 2016). These molecular changes are thought to underlie disruptions in normal developmental dendritic pruning, as adult brains with FXS contain longer dendritic spines and more synaptic connections than average in both humans and mouse models (Grossman et al., 2006; Patel et al., 2014). In multiple animal models, loss-of-function mutations of FMRP cause an increase in terminal dendritic branches, and in *Drosophila*, overexpression of FMRP has been found to decrease dendritic branch number (Comery et al., 1997; Lee A. et al., 2003; Pan et al., 2004; Zhang and Broadie, 2005; Xu et al., 2008; Dahlhaus, 2018; Khayachi et al., 2018; Wang X. et al., 2018).

Disinhibition of Arc, causing over-active translation, may underlie the excess of synaptic connections found in *fmr1* knock out brains. In rat pyramidal neurons, ∼13% of proteins enriched in “excitatory” synaptic terminals (those containing metabotropic glutamate receptors [mGluRs]) were definite targets of FMRP (Hafner et al., 2019). However, mGluRs are also involved in long term depression (LTD) of synapses. LTD requires well-timed protein synthesis in order to occur. Arc must be translated within five to ten minutes of experience for the excitatory AMPA receptors to be endocytosed and LTD to occur, but if Arc is constantly translated, its ability to signal LTD is extinguished (Park et al., 2008). Homeostasis is therefore attacked on two fronts: by the increased translation of proteins that encourage dendritic growth and synapse formation, and by the inability of the LTD process to naturally remove excess synapses. It is still unknown how the increase in branching and synaptic connections leads to the cognitive symptoms of FXS or what other, less visible consequences the overactive ribosomes may have on the cell. The widespread impact of the loss of FMRP – one ribosomal-associated protein – is a testament to the importance of protein synthesis in dendrites, only the first arm of the proteostasis system.

### Protein Maintenance

Once proteins are synthesized, they commute to their work sites via the secretory pathway or are simply freed into the cytoplasm to carry out their duties. Post-translational modifications are largely responsible for adaptive protein responses to cell conditions and include acetylation, phosphorylation, methylation, SUMOylation, ubiquitination, ISGylation, nitrosylation, and ROS generation (Ren et al., 2014; Sambataro and Pennuto, 2017). All of these post-translational modifications are integral to dynamic control of protein activity, but are outside of the scope of this review. The protein maintenance section will chiefly focus on the role of chaperones in dendrites.

**FIGURE 2:**
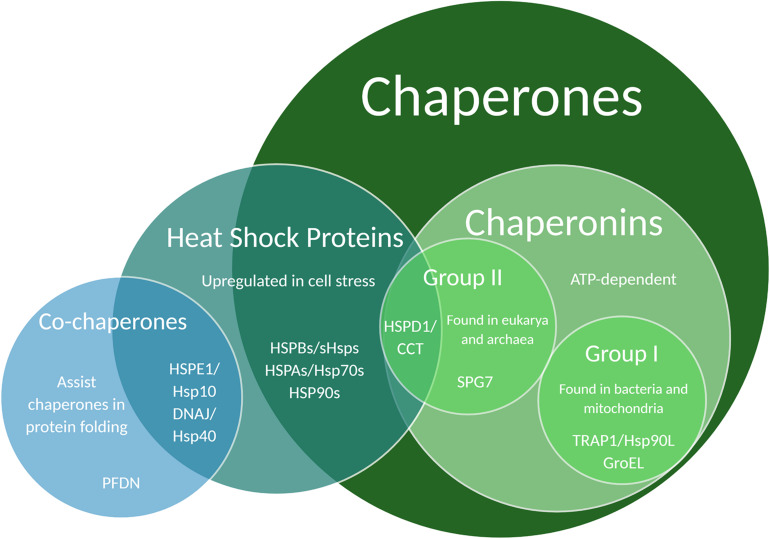
Venn diagram illustrating chaperone and co-chaperone families. Specific chaperones listed in this Venn diagram have been associated with regulatory effects on dendritic development and function.

Protein structure is integral to function, thus a key component of protein maintenance is the modulation of protein folding through the action of chaperones. Chaperones are proteins that help other proteins to fold correctly. They can be found in the cytosol as well as organelles like the ER and mitochondria (Benitez et al., 2014; Brehme et al., 2014). Many chaperones are also heat shock proteins (Hsps) because of their upregulation during times of heat stress, and are commonly categorized by weight – an Hsp60 chaperone is approximately 60 kiloDaltons (kDa) (Garrido et al., 2012). A small subset of chaperones require ATP to function and are called chaperonins (Buxbaum, 2015). Chaperonins are further split into Group I – found in bacteria and mitochondria – and Group II – found in eukarya and archaea (Balchin et al., 2018; Figure 2). Chaperonins are capable of encompassing misfolded proteins and encouraging unfolding and refolding through chaperonin conformational cycling and hydrophobic residue interactions, whereas ATP-independent chaperones cannot themselves refold a protein, but can bind it to prevent it from damaging interactions with other proteins (Narberhaus, 2002). Because of this difference, chaperonins and ATP-independent chaperones are occasionally referred to as “foldases” and “holdases,” respectively (Garrido et al., 2012; Bakthisaran et al., 2015; Ali et al., 2016; Penke et al., 2018; Hipp et al., 2019).

### Protein Maintenance in Dendritic Arborization

Protein maintenance is critical for neuronal processes: misfolded proteins must be cleared quickly before they cause damage and are either refolded or replaced. Chaperones are required from the beginning of dendritic development, as can be seen, for example, by disruptions in neurite differentiation under Hsp70 or Hsp90 knockdown conditions (Benitez et al., 2014; Miller and Fort, 2018). Hsc70, a non-heat-inducible form of Hsp70 may also be necessary for neurite differentiation. Mutations in the BAG2-Hsc70 chaperone complex was found to cause synaptic vesicles, normally located in the axon, to appear in the spines and processes of dendrites (Fukuzono et al., 2016). Chaperones contribute a great deal to cytoskeletal stability, which is especially important for neurons to sustain their complex processes (Table 2; Bakthisaran et al., 2015; Nefedova et al., 2015; Nefedova et al., 2017; Kelliher et al., 2019; Muranova et al., 2019; Vallin and Grantham, 2019; Muranova et al., 2020). Many chaperones are implicated specifically in axonal morphology: manipulation of several small heat shock proteins as well as Hsp70 and 90 was found to significantly decrease synapse number in *Drosophila* neuromuscular junctions (Santana et al., 2020), and an Hsp70 orthologue has been found to assist in polarized trafficking of synaptic vesicle proteins to axons (Fukuzono et al., 2016).

**TABLE 2:** *Protein Maintenance Dendritic Phenotypes* proteins involved in regulating protein maintenance cause a variety of dendritic phenotypes when manipulated.

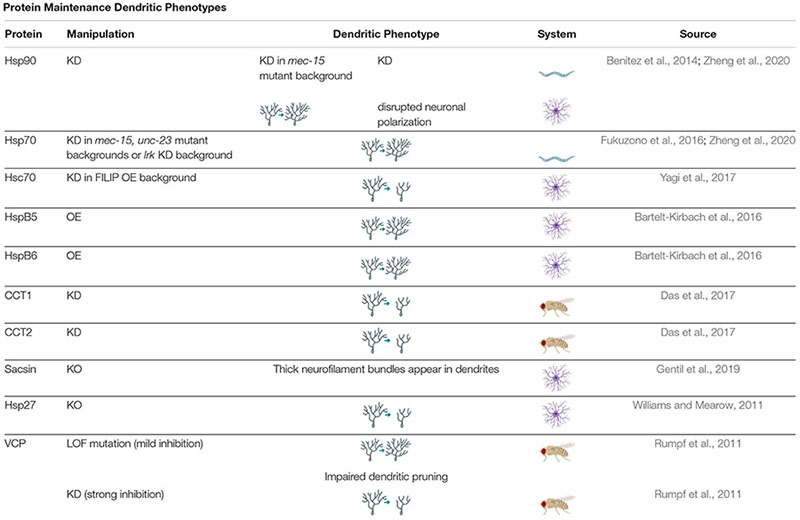

While some chaperones have only been reported to affect axon morphology, others have been reported to affect only dendrites (Bartelt-Kirbach et al., 2016). The small heat shock proteins, Hspb5 and Hspb6, were found to increase dendritic complexity without altering axonal morphology when overexpressed in rat hippocampal neuron culture (Bartelt-Kirbach et al., 2016). Hspb5 and Hspb4, better known as αB-crystallin and αA-crystallin, make up the majority of lens protein in vertebrate eyes, and have both been found to stabilize the cytoskeleton – though Hspb4 has only been found to associate with intermediate filaments in rat retinal glia but not neurons (Bakthisaran et al., 2015; Zayas-Santiago et al., 2018).

The differing function between two such closely related chaperones is exemplary of the diversity of the small Hsp family. The small Hsp family is, ironically, one of the largest families of chaperones, with ten members in humans – HSPB1-10 – that all have diverse protein clients and are expressed in an array of tissues throughout the body, though most are expressed in the brain as well (Carra et al., 2012; Mymrikov et al., 2020). Traditionally thought of as chaperones that act as holdases to prevent protein aggregation in cell stress conditions, it has been discovered that several small Hsps contribute to proper dendritic arborization in homeostatic conditions (Narberhaus, 2002; Bakthisaran et al., 2015). Several small Hsps were found to physically interact with neurofilaments *in vitro* (Nefedova et al., 2017), and Hspb3 was found to distribute along neurofilaments in axons in mouse and chicken spinal motoneurons *in vivo* (La Padula et al., 2016). The phosphorylated forms of Hspb1 and Hspb5 associate with filamentous structures in both axons and dendrites, and mutations of Hspb1 have been found to cause cytoskeletal abnormalities, both *in vitro* and *in vivo* in mouse neurons (Schmidt et al., 2012; Lee et al., 2015; Sarparanta et al., 2020), though traditionally Hspb1 is associated with the axonal abnormalities underlying Charcot-Marie-Tooth disease (Miller and Fort, 2018; Muranova et al., 2019). More work needs to be done to uncover the role of small heat shock proteins in cytoskeletal maintenance and dendritic arborization.

Many other chaperones support dendritic development through maintenance of the cytoskeleton (Zheng et al., 2020). For example, the Hsp60 Chaperone Containing Tailless complex polypeptide-1 (CCT), is required to fold β-tubulin and actin (Brackley and Grantham, 2009; Sergeeva et al., 2014). In *Drosophila* CIV neurons, knockdown of CCT subunits CCT1 and 2 caused dendritic arbors to develop abnormally, with simplified, smaller arbors that contained significantly less F-actin and microtubules than controls (Das et al., 2017). Similarly, knockdown of *Prefoldin 5* (*Pfdn5*), a component of the prefoldin co-chaperone complex, which is known to assist CCT in folding, also leads to reduction in microtubule density in CIV neurons (Tang et al., 2020). Hsp70 is also required for the development of lasting, stable dendritic arbors because it folds free tau, which is important for the stabilization of microtubules (Abisambra et al., 2013). Finally, sacsin, an enormous 520 kDa protein, has just been classified in the past decade as a chaperone because of its Hsp90 and DNAJ (Hsp40)-like domains which may function as chaperone and co-chaperone, respectively (Anderson et al., 2011). Sacsin is required for proper organization of neurofilaments in several types of neurons, including Purkinje and pyramidal neurons, and when mutated – as in the case of the hereditary disorder autosomal recessive spastic ataxia of the Charlevoix-Saguenay – leads to abnormal somatodendritic bundles of neurofilaments (Gentil et al., 2019; Larivière et al., 2019). The localization and mutation studies clearly indicate an important role of chaperones in forming and maintaining dendritic arbors.

### Protein Maintenance in Cell-Type Specificity

Levels of chaperones fluctuate from tissue to tissue, as best exemplified through the small Hsp family. Hspb4 and 5 are found in high levels in the vertebrate eye lens, for example, whereas Hspb9 is only found in the testis (Mymrikov et al., 2011; Garrido et al., 2012). In a *C. elegans* screen of chaperones, Hsp70 showed broad expression, but a mitochondrial Hsp70 showed high expression in the intestine (Guisbert et al., 2013). Furthermore, chaperone level differences exist between cell types within the brain. In rat brain and spinal cord slices, Hsc70, a non-heat-dependent form of Hsp70, was found at higher rates in dopaminergic and motor neurons than in entorhinal cortical or hippocampal neurons (Chen and Brown, 2007). The variable levels of chaperones might mean that different cell types have variable reliance on their protein folding activities. For example, sacsin deficits have been found to cause more distinct neurofilament accumulations in certain cell types, and *sacsin* knockout mice displayed progressive cell loss of Purkinje neurons in the anterior cerebellar lobules at significantly greater levels than in Purkinje neurons in other cerebellar regions (Ady et al., 2018; Larivière et al., 2019). The observed differences could be due to higher cytoskeletal demands from neurons with complex arbors and thus greater sensitivity to cytoskeletal disorganization, and it has been speculated that the region-specific vulnerability of Purkinje neurons could be due to intrinsic cell qualities that differ from region to region, such as average firing rate (Ady et al., 2018; Larivière et al., 2019). However, cell-dependent reliance on chaperones could also be explained by chaperone-chaperone interactions or non-canonical homeostatic functions of chaperones.

Chaperone-chaperone interactions may contribute to cell-type differentiation. Chaperones can overlap in their “clientele,” as in the case of Hsp70 and CCT, which share seventy client proteins in common (Aswathy et al., 2016). Hsp70 has also been found to deliver clients to CCT and the two chaperonins have been shown to coordinate in folding large, multidomain proteins (Kim et al., 2013). In rat hippocampal cell culture, inhibition of Hsp90 led to Hsc70 re-localization from its usual subcellular location in proximal processes to the soma and distal axonal processes, where Hsp90 is normally found (Benitez et al., 2014), indicating that Hsc70 may change its location to compensate for the absence of Hsp90.

Small heat shock proteins are known to group together in hetero- and homo-oligomers, although the function of these oligomers is not yet known (Nefedova et al., 2015; Mymrikov et al., 2020). The interaction of chaperones may be the key to regulation of neuronal processes. Hspb6 has been found to act as a modulator of activity for other small Hsps in human cell culture (Weeks et al., 2018; Mymrikov et al., 2020; Santana et al., 2020). Furthermore, individual overexpression of Hsp23 or Hsp26 led to decreased number of synapses in the *Drosophila* neuromuscular junction, but combined overexpression of Hsp23 and Hsp26 together resulted in an increase in synapse numbers. From these surprising results and additional interactions with the novel kinase *Pinkman*, the authors hypothesize that Hsp23 and Hsp26 form a complex promoting synaptic formation and that the imbalance of the two chaperones may be the cause of synaptic dysregulation, rather than loss or gain of each individually (Santana et al., 2020).

Chaperones may also help regulate dendritic arborization in a manner apart from their canonical protein folding function. Many heat shock proteins have been found to have “moonlighting” roles in addition to their canonical protein folding duties (Jeffery, 2018). Some of these occur in separate environments, such as in the case of Hsp60 which acts as a mitochondrial chaperonin inside the cell, but as an Apo-lipoprotein A receptor on the membranes of human cultured hepatocytes (Jeffery, 2018; Bocharov et al., 2000). Some Hsps are even thought to be secreted from cells as anti-inflammatory agents (Edkins et al., 2018). Crystallins, long known for their chaperone activities in the lens of the eye, have also been found to have enzymatic functions (Fares, 2014; Jeffery, 2018).

Hsc70 performs both its canonical and moonlighting functions within the cytoplasm. In addition to its protein folding activities, Hsc70 has been found to bind to filamin-A interacting protein, which in turn binds to myosin IIb, an actin-binding protein which helps to regulate the shape a dendritic spines – in effect, Hsc70 promotes dendritic spine elongation through this pathway (Yagi et al., 2017). Additionally, the chaperone function of Hsp27 has been found to be dependent on its phosphorylation state which impacts its ability to form large homo-oligomers made up of other Hsp27 proteins. In Hsp27 knockout and phosphomimetic conditions, cultured rat neurons grew significantly fewer neuritic processes, implicating the phosphorylation state of Hsp27 in axonal and dendritic arbor formation (Williams and Mearow, 2011). Phosphorylation also affects the subcellular localization of Hspb1 and Hspb5, which moved from the soma to the dendrites and neuronal processes, respectively, when phosphorylated (Schmidt et al., 2012). This is especially interesting given that phosphorylated forms of small heat shock proteins have been shown to be less effective as chaperones in *in vitro* studies (Schmidt et al., 2012), supporting the idea that chaperone moonlighting may contribute to cell-specific support of diverse dendritic arbors.

### Protein Maintenance in Cell Stress

Because of the dependence on cytoskeletal proteins for the maintenance of their dendritic arbors, there are some indications that neurons with large dendritic arbors may be particularly susceptible to the effects of cell stress, particularly heat shock (Dalle-Donne et al., 2001; Klose and Robertson, 2004). Without properly organized microtubules, actin, and neurofilaments, the dendritic and axonal arbors cannot be maintained (Kelliher et al., 2019). Cell stress has been shown to prevent proper maintenance of dendritic arbor and spine morphologies (Klose and Robertson, 2004; Nie et al., 2015), and in *C. elegans*, heat shock in adolescence has been shown to alter neuronal morphology (Hart, 2019). Therefore, chaperone-mediated maintenance of the dendritic arbor is especially important in times of neuronal stress.

Many, but not all chaperones are Hsps, named for their upregulation during heat stress. The increased numbers of Hsps is thought to combat higher levels of misfolded proteins during cell stress, and promote cell health (Miller and Fort, 2018). Hsps can be neuroprotective against cell death, as in the case of members of the Hsp70 family (HSPA1A and HSPA6), which when knocked down decreased cell viability in differentiated human neuronal cells undergoing heat shock (Deane and Brown, 2018). Upregulation of Hsps can also rescue morphology, as in the case of small Hsp23. When Hsp23 was overexpressed *Drosophila* muscle cells, it was able to prevent heat shock-induced axonal degeneration of the connected motor neurons (Kawasaki et al., 2016). The presence of Hsps during heat shock can even protect against dysregulation of cell dynamics. For example, boutons in the *Drosophila* neuromuscular junction fail to release neurotransmitter during heat shock, but overexpression of Hsp70 can rescue boutons and enable neurotransmitter release to continue (Klose and Robertson, 2004). It must be noted that these examples are all involving axons and uncategorized neurites, and the neuroprotective effects of Hsps in dendrites is in need of further research.

Heat shock is not the only type of cellular stress that can upregulate Hsp expression (Mymrikov et al., 2011; Schmidt et al., 2012; Chaari, 2019; Hu et al., 2019). Hsp70, for example, has been used as a marker of cell stress in epilepsy (Hu et al., 2019), and the upregulation of Hsp70 in these conditions is not without good reason: high expression of Hsp70 has been found to be neuroprotective in rat motoneurons experiencing excitotoxicity (Shabbir et al., 2015). Other neuronal stressors leading to the activation of heat shock proteins can include oxidative stress (Mymrikov et al., 2011; Fukui et al., 2019), hypoxia (Schmidt et al., 2012), and ER stress (Ryoo et al., 2007; Xu et al., 2011).

ER stress can induce apoptosis, and even under mild conditions of ER stress, neurite differentiation and dendritic length are disrupted (Kawada et al., 2014). As mentioned in section “Protein Synthesis Linked to Neurological Disease,” the UPR is an adaptive cell response to ER stress, which in part involves the triggering of IRE1 and subsequent activation of XBP1. XBP1 activation was found to upregulate ER DnaJ/Hsp40 (a co-chaperone to Hsp70) expression in mouse fibroblasts undergoing ER stress, and another UPR factor, ATF6, is thought to recruit BiP (an ER-specific Hsp70) in times of ER stress (Lee A.-H. et al., 2003). These ER chaperones and co-chaperones are necessary to stave off the high levels of ER stress which can lead to apoptosis. Another ER chaperone, valosin-containing protein (VCP), was found to be necessary for dendritic pruning in *Drosophila* (Rumpf et al., 2011). Mild inhibition of VCP caused ER stress and suppressed developmental dendritic pruning, while strong VCP inhibition caused severe dendritic morphology defects and cell death (Rumpf et al., 2011). It is possible that the connections between ER stress and chaperone response are not only neuroprotective defenses against apoptosis, but perhaps part of a larger molecular cascade regulating the dendritic arbor (Martínez et al., 2018).

### Protein Maintenance in Disease

The ability of chaperones to refold misfolded proteins and disassemble protein aggregations implicates them in most proteinopathic diseases, as well as neuropathies. In select cases, mutations in the chaperone proteins themselves appear to contribute to disease etiology. For example, mutations in *HSPB1*, *3*, and *8* are associated with Charcot-Marie-Tooth Disease (Mymrikov et al., 2011) and mutations in *HSPB8* are also associated with hereditary spastic paraplegic neuropathy (Mymrikov et al., 2011), two diseases associated with axonal degeneration. Subunits four and five of the CCT complex have also been causatively linked to hereditary spastic paraplegic neuropathy; mutations in subunit five (CCT5), specifically, are the most likely candidates in causing mutilating hereditary sensory neuropathy with spastic paraplegia in a Moroccan family with the condition (Bouhouche et al., 2006; Sergeeva et al., 2014; Pavel et al., 2016). It is interesting to note that although the CCT mutations are body-wide, the effects specifically manifest in motor neurons – this, despite the fact that CCT is ubiquitously expressed in all tissues, not concentrated in neural tissue (Mymrikov et al., 2011; Guisbert et al., 2013; Hu et al., 2019). Although these examples are of mutations which cause symptoms to arise because of degeneration of the axon, there are less obvious disease links to dendritic arbor malformation. For example, in a genetic screen of mutations associated with schizophrenia and ASD, 16% of the genes screened were found to be required for proper dendritic morphology in *C. elegans* (Aguirre-Chen et al., 2020). Additionally, though the symptoms of autosomal recessive spastic ataxia of the Charlevoix-Saguenay are mainly due to axonal deformities, the underlying mutation of the sacsin chaperone also results in neurofilament bundling in dendrites (Anderson et al., 2011; Gentil et al., 2019; Larivière et al., 2019). The effects of dendritic arborization deficits in these rare diseases may have been previously overshadowed by the dramatic axonal effects, and should be examined in future studies.

Disruptions in protein maintenance also seem to predispose brains to neurodegenerative disease. For example, protein maintenance machinery has been found to decrease in aged brains (Brehme et al., 2014), and has thus been implicated in age-related proteinopathies such as AD and PD. CCT levels are depressed in AD patients and select subunits have also been found to be under-expressed in brains with Down syndrome, a condition known to be highly correlated with early onset AD (Aswathy et al., 2016). CCT has also been shown *in vitro* to inhibit the assembly of α-synuclein – as have Hsp70 and some Hsp40 co-chaperones (Aprile et al., 2017; Sot et al., 2017). α-synuclein is the aggregating protein found in the Lewy bodies of some neurodegenerative diseases such as PD (Sot et al., 2017). Though traditionally reported to aggregate in the soma and axons of neurons, it was recently found that expression of human α-synuclein in mouse cortical neurons localized to the soma and dendrites of Layer V cortical neurons and caused them to show increased dendritic spine density (Lim et al., 2020; Wagner et al., 2020). This finding runs counter to previous work stating that overexpression of α-synuclein in the cortex causes dendritic spine loss and dendritic arbor malformation in Layer V neurons (Blumenstock et al., 2017). However, since these studies were performed in younger and older mice, respectively, the findings together could indicate age-dependent dendritic effects of α-synuclein expression, perhaps underlying some of the symptoms of PD. Hsc70, Hsp27 and the mitochondrial chaperone, TRAP1, are also implicated in different hereditary forms of PD (Fukuzono et al., 2016; Brunelli et al., 2020; Vicente Miranda et al., 2020).

Chaperones are of particular interest to those studying proteinopathies and their devastating neurological effects (Smith et al., 2015). Protein aggregations are thought to be particularly for neurons because of the demands of maintaining axonal and dendritic arbors (Lim and Yue, 2015), and neurodegenerative diseases usually cause changes in dendritic morphology (Penke et al., 2018). Hsp70 has been found to co-localize with Ab plaques (Broer et al., 2011). Co-chaperones have also been explored for their contribution to proteostasis in disease. DNAJB6, an Hsp40 co-chaperone, was recently found to help regulate ataxin poly Q aggregates (Molzahn and Mayor, 2020; Thiruvalluvan et al., 2020).

CCT is widely reputed to associate with, disaggregate, or otherwise reduce toxicity of mutant huntingtin protein aggregates (Tam et al., 2006; Brackley and Grantham, 2009; Aswathy et al., 2016; Chen et al., 2018). A recent study has confirmed that CCT works to prevent formation of mutant huntingtin aggregates in adult mouse neural progenitor and stem cells (NPSCs), but more excitingly, the research implicates a balance of chaperones that work to attenuate the damages of aggregations in multiple ways over the course of cell differentiation and development. It was discovered that CCT and small Hsp levels are inversely regulated – CCT more highly expressed in NPSCs and Hspb5 more highly expressed in differentiated neurons. CCT works in the prevention of aggregates in NPSCs, but Hspb5 works in the sequestration of aggregates in differentiated neurons: all of this indicating that neurons may have developmentally regulated responses to protein aggregation, delineated by “foldase” and “holdase” chaperone properties (Molzahn and Mayor, 2020; Vonk et al., 2020).

Given their associations, upregulation of chaperones could prove to be neuroprotective, and chaperones have been targeted as potential neuroprotective agents for therapeutic interventions. There has been a recent boom in the literature of chaperone-as-medicine techniques – in 2018 the *Philosophical Transactions of the Royal Society B* devoted an entire themed issue to “heat shock proteins as modulators and therapeutic targets of chronic disease” (Edkins et al., 2018). Although generally the upregulation of chaperones has been found to be beneficial in fighting neurodegenerative diseases (Smith et al., 2015), counterintuitively, loss-of-function experiments with CCT subunits as well as an Hsp40 member was found to be neuroprotective in a *C. elegans* model of Aβ toxicity (Khabirova et al., 2014). Similarly, application of 17-AAG, which inhibits Hsp90, rescued dendritic spine loss from Aβ-induced degradation in mice (Chen et al., 2014; Smith et al., 2015). Despite these findings, the literature generally points towards a neuroprotective effect of chaperone overexpression, so several avenues have been explored in using chaperones to alleviate neurodegenerative disease (Dar et al., 2020). In primary neuronal mouse cultures with human tau mutations, application of YM-01 was used to chemically induce Hsc70 affinity for free (non-microtubule-bound) tau, leading to lower levels of the free tau, which can cause tangles within the cytoplasm (Abisambra et al., 2013). Applications of 1,4-dihydropyridine derivatives have also been used in attempts to increase levels of heat shock proteins by inducing cellular stress in mouse models of Alzheimer’s (Kasza et al., 2016). Unfortunately, the side effects of pharmacologically stimulating chaperones *en masse* in the brain are likely to be numerous. Hsp90 antagonists like geldanamycin have been used in clinical trials for treatment of cancer, with subjects reporting side-effects such as pain and fatigue, which may be explained by work showing that geldanamycin impairs neurite growth and cytoskeletal maintenance (Migita et al., 2019). So far, no “chaperonotherapies” have been approved for treatment of neurodegenerative diseases. However, Arimoclomol, a drug which stimulates expression of Hsp70, recently came through Phase II human clinical trials for Amyotrophic Lateral Sclerosis (ALS) with encouraging results, and is on schedule to finish Phase III trials by 2021 (Lanka et al., 2009; Elliott et al., 2020; Orphazyme’s arimoclomol receives US Fast Track Designation in Amyotrophic Lateral Sclerosis, 2020).

### Protein Degradation

The final arm of the protein quality control system is protein degradation. Cellular homeostasis is achieved in part through the Ubiquitin Proteasome System (UPS), the endosome-lysosome (endolysosomal) degradation pathway, and autophagy, which are involved in protein degradation and are important not only for ridding the cell of unsalvageable misfolded proteins, but also for producing new materials by recycling protein components (Clark et al., 2018; Gerónimo-Olvera and Massieu, 2019). There is high crossover and cooperation between these systems: all three rely on ubiquitination, and all three conventionally end in fusion with a lysosome, though autophagosomes and endosomes can also fuse during trafficking (Tooze et al., 2014; Jin et al., 2018).

The UPS is used by cells to clear soluble proteins from the cytoplasm; these can be misfolded, malfunctioning proteins or even correct conformations that are simply not needed in the cytosol at the time (Ciechanover and Kwon, 2017; Kocaturk and Gozuacik, 2018). The unwanted proteins are ubiquitinated, or tagged with small ubiquitin molecules, through a process involving several enzymes (E1 through E3) (Hamilton and Zito, 2013; Kocaturk and Gozuacik, 2018). E1 is known as a ubiquitin activating enzyme, and is the enzyme that uses ATP to “activate” a ubiquitin tag. E1 then passes the ubiquitin onto E2. E2 transfers the ubiquitin to the E3 ligase – already bound to the protein target – and finally the E3 ligase transfers the ubiquitin tag to the target protein itself (Hamilton and Zito, 2013; George et al., 2018). This cycle may be repeated and more ubiquitin tags added to the first. The ubiquitinated proteins are then degraded by lysosomes or proteasomes or otherwise regulated depending on the organization of the polyubiquitination (Hamilton and Zito, 2013). Endolysosomal degradation also begins with ubiquitination, though normally of membrane proteins, which are then endocytosed and fused with early endosomes. Early endosomes then mature into multivesicular bodies, then finally to late endosomes which fuse to lysosomes (Jin et al., 2018; Zhang et al., 2014).

Autophagy, as in the other two degradative pathways, often begins in ubiquitination. However, the final location of proteins varies between UPS, endolysosomal degradation, and autophagy: UPS-mediated degradation results in proteasomes breaking down proteins, while in autophagy and endolysosomal degradation, lysosomes are often responsible for recycling the waste (Kocaturk and Gozuacik, 2018). Additionally, UPS is a highly specific system that primarily degrades single cytosolic proteins, whereas autophagy can degrade a wider variety of cytosolic substances as well as membrane proteins, which, as previously mentioned, are also degraded via endolysosomal pathways (Jin et al., 2018; Kocaturk and Gozuacik, 2018; Gerónimo-Olvera and Massieu, 2019). Since this can involve breakdown of organelles, proteins, or parasitic invaders, there are many selective forms of autophagy named for their targets, such as mitophagy (mitochondrial autophagy) and even proteaphagy (proteasome autophagy) (Yang and Klionsky, 2009; Yang et al., 2013; Kocaturk and Gozuacik, 2018; Fang et al., 2019).

There are three main types of autophagy: macro-, micro-, and chaperone-mediated autophagy (Yue et al., 2009; Gerónimo-Olvera and Massieu, 2019). In general, autophagy is the process by which cytosolic matter is transported to lysosomes to be broken down. Macroautophagy involves the creation of autophagosomes, which engulf the targets in membrane and contain them during transport to the lysosome (Maruzs et al., 2019).

Chaperones can also capture and transport misfolded proteins to the lysosome in a process called chaperone-mediated autophagy (Ciechanover and Kwon, 2017). Hsp70 is known to mediate disassembly of protein aggregates in this manner (Fukuzono et al., 2016; Ciechanover and Kwon, 2017). Chaperones not only can correct misfolded proteins but can disassemble aggregations and initiate UPS and autophagic pathways (Mymrikov et al., 2011; Fukuzono et al., 2016; Kasza et al., 2016; Pavel et al., 2016; Ciechanover and Kwon, 2017; Chaari, 2019). While they may or may not participate in physically transporting misfolded proteins to lysosomes, other chaperones - including CCT, Hspb6, and Hspb8 - are implicated in positively regulating autophagy (Mymrikov et al., 2011; Pavel et al., 2016).

Finally, microautophagy occurs when the lysosomes directly capture and break down cytosolic content (Yue et al., 2009). This wide variety of autophagic sub-systems is fascinating, and each component is individually important for proteostasis. However, generally, the word autophagy refers to macroautophagy, and that is how the term will be used for the remainder of this review.

### Protein Degradation in Development and Maintenance of Dendritic Arbors

Protein degradation, like the other arms of protein quality control, is crucial for dendritic arbor formation and maintenance. Neurons are post-mitotic and thus cannot clear cellular waste through division (Son et al., 2012; Maday et al., 2014; Ciechanover and Kwon, 2017). Additionally, autophagy is especially important for degradation of long-lived proteins (Kocaturk and Gozuacik, 2018), which are often found in the brain (Alvarez-Castelao and Schuman, 2015) and membrane proteins, which are integral to the function of neuronal synapses (Hamilton and Zito, 2013).

Loss-of-function studies of components of the UPS have been found to disrupt dendritic development. Knockdown of Ube3A, an E3 ligase, was found to reduce dendritic arborization in *Drosophila* neurons, and mutations of Ube3A are associated with Angelman Syndrome (AS), discussed in section “Protein Degradation and Disease” (Lu et al., 2009; Hamilton and Zito, 2013). Knockdown of *SkpA*, an adaptor protein of the SCF (Skp-1-Cullin-F-box) E3 ubiquitin ligase complex, causes increased branching in *Drosophila* larval CIV neurons, and is later implicated in CIV neurons for regulating dendritic pruning at the pupal stage, discussed more in section “Protein Degradation in Neuronal Dynamics” (Wong et al., 2013; Das et al., 2017; Nanda et al., 2018).

Distinctions have been noted in the mechanisms and activity of protein degradation between axons and dendrites, and these differences may be important for developing and maintaining compartmentalization in the neuron. For example, the Anaphase Promoting Complex (APC) is a large E3 complex that ubiquitinates target proteins for UPS, and has been found to negatively regulate axonal growth through activation of downstream transcription factors; however, APC was also found to positively regulate dendritic growth and arborization (Konishi et al., 2004; Kim et al., 2009; Hamilton and Zito, 2013). Highwire, another E3 ubiquitin ligase has been found to have similar regulatory capabilities (Table 3; Wang et al., 2013). There are also differences in autophagosome transport in axons and dendrites that indicate compartmentalization of autophagosome activity. Autophagosomes are often formed in axons before being transported to the soma (Yue et al., 2009; Hernandez et al., 2012; Maday and Holzbaur, 2016). In primary mouse hippocampal cell culture, 80% of all counted autophagosomes were formed in the distal tip of the axon before moving retrograde to the cell body, where they were held before forming autolysosomes. Meanwhile, dendritic autophagosomes primarily remained stationary, and the few that moved did so bidirectionally on the dendritic arbor (Maday and Holzbaur, 2014). Based on these patterns of movement, and common mixed polarity microtubule organization in mammals, it appears that autophagosome transport is dependent on microtubules and retrograde motors, such as dynein (Yang et al., 2013; Maday and Holzbaur, 2014, 2016). Endosomes, which also appear to depend on dynein for transport, have been found to traffic bidirectionally in dendrites, as have lysosomes (Satoh et al., 2008; Jin et al., 2018; Yap et al., 2018). The movement of endosomes is also dependent on the small GTPase Rab11 (Park et al., 2006). Furthermore, locations of lysosomes, endosomes, and autophagosomes throughout the dendritic arbor differ. For example, early endosomes were found to be abundant in the medial and distal dendrites of rat hippocampal neurons, whereas lysosomes tended to concentrate closer to the soma in the proximal dendrites (Yap et al., 2018). The small GTPase Rab5, a component of the early endocytic pathway, has been shown to associate with cytoplasmic Dynein to promote dendritic branching (Satoh et al., 2008). Furthermore, components of the endosomal sorting complex required for transport (ESCRT) machinery have been implicated in regulating dendritic growth (Sweeney et al., 2006; Firkowska et al., 2019). Finally, the recycling endosome marker Rab11 has been implicated in proper dendritic arbor formation in *Drosophila* larvae and rat hippocampal neurons (Krämer et al., 2019; Siri et al., 2020).

**TABLE 3:** *Protein Degradation Dendritic Phenotypes* proteins involved in regulating protein degradation cause a variety of dendritic phenotypes when manipulated.

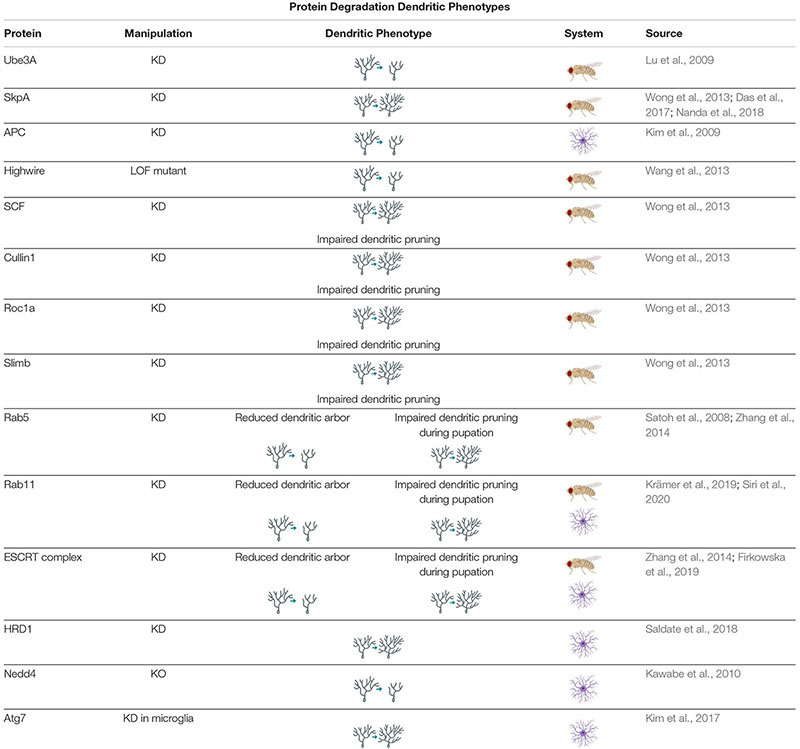

There is also a lack of consensus as to whether an increase in autophagic activity positively or negatively regulates dendritic growth (Son et al., 2012). In *Drosophila*, knockdown or overexpression of autophagic components leads to simplification or increased branching, respectively, in the axonal neuromuscular junction (Shen and Ganetzky, 2009; Hernandez et al., 2012). However, in *Drosophila* sensory neurons, both knockdown and overexpression of autophagy (*Atg*) genes results in simplified arbors, which indicates that proper dendritic development requires carefully modulated levels of basal autophagy in homeostasis (Clark et al., 2018). In mammals, decreased autophagy can lead to increased spine density but a decrease in total dendritic length via shortening of the apical dendrite in mouse dentate gyrus neurons (Schäffner et al., 2018) – whether that is an overall increase or decrease in arbor complexity is unclear. When the autophagy of mitochondria – known as mitophagy – is manipulated in neurons, it can have effects on dendritic growth, though in what way depends on the stage of neuronal development (Brot et al., 2014).

The mixed findings may be partially due to experimental methods of autophagic induction. A common method of inducing autophagy is rapamycin, which inhibits mTOR (mammalian target of rapamycin) activity, thus disinhibiting autophagy and increasing the number of autophagosomes (Hernandez et al., 2012; Yang et al., 2013). However, the mTOR complex phosphorylates multiple kinases as well as transcription and growth factors, and inhibition of this complex may have off-target effects (Mitra et al., 2009; Lamming, 2016). Additionally, there are many studies which use starvation or other methods of induction that are not specific to just autophagy in order to examine the effects of autophagy (Brot et al., 2014; Gerónimo-Olvera and Massieu, 2019; Zhou et al., 2019). For example, tunicamycin was used to induce ER stress and, indirectly, autophagy in an experiment designed to examine the effects of autophagic flux on dendritic arbors in primary rat neuronal culture (Zhou et al., 2019). While these methods do increase autophagic action, it may be that there is no consensus on the effects of autophagic disruption in axonal and dendritic arbors because of the diversity of autophagic induction methods and the varying off-target effects of those methods (Son et al., 2012; Maday et al., 2014; Clark et al., 2018; Gerónimo-Olvera and Massieu, 2019).

### Protein Degradation in Neuronal Dynamics

Protein degradation systems are required in neurons to form proper dendritic arbors. One particular way in which UPS contributes to the dynamic dendritic arbor is through pruning (Yu and Schuldiner, 2014). Pruning is a developmentally timed removal of dendritic and axonal branches, such as in humans during childhood or in *Drosophila* in preparation for metamorphosis (Tang et al., 2014; Yu and Schuldiner, 2014); however, similar processes can also facilitate the removal of dendritic processes and spines as a response to LTD (Piochon et al., 2016).

UPS, autophagy, and the endolysosomal pathway are required for developmental dendritic pruning, and thus for final dendritic arbor shape (Hamilton and Zito, 2013; Yu and Schuldiner, 2014). UPS inhibition leads to defects in dendritic severing, and both DIAP1 (a *Drosophila* E3 ubiquitin ligase) and SCF (another E3 ubiquitin ligase) are required for developmental dendritic pruning in *Drosophila* (Rumpf et al., 2011; Wong et al., 2013; Yu and Schuldiner, 2014). Autophagy, too, is required for developmental dendritic pruning: knockout of *Atg7* – which has also been reported to selectively disrupt axonal formation – reduced spine elimination without affecting spine formation in mouse hippocampal cell culture (Tang et al., 2014). The SCF E3 ubiquitin ligase complex, comprised of cullin1, Roc1a, SkpA, and slimb is required for dendritic pruning of *Drosophila* CIV neurons at the pupal stage whereas knockdown of *SkpA* results in supernumerary growth and branching of CIV dendritic arbors at the larval stage (Wong et al., 2013; Kanamori et al., 2015a; Das et al., 2017; Nanda et al., 2018). Finally, the endolysosomal pathway also affects dendritic pruning and arbor shape. Loss of Rab5 and ESCRT proteins cause dendritic pruning defects through disrupting the endocytosis of the cell adhesion molecule, neuroglian (Zhang et al., 2014). It has also been found that Rab5 and dynamin – two more GTPases involved in the endolysosomal pathway – are required not only for the dendritic thinning that precedes detachment during developmental pruning in *Drosophila* CIV neurons, but also for the calcium transient currents that appear during the pruning process. The mechanism by which this occurs is still unknown, but the finding indicates that the endolysosomal pathway is not simply clearing dendritic membrane to promote pruning, but may also be actively involved in the signaling pathway that determines which processes are removed (Kanamori et al., 2015b).

Ubiquitin-proteasome system and autophagy have been well-established as an integral part of LTD (Hamilton and Zito, 2013; George et al., 2018; Mabb and Ehlers, 2018), but the mechanisms are still unknown. There are some studies which indicate that dendritic autophagy and UPS are required for NMDA-mediated LTP through removal of AMPA receptors (Lin et al., 2011; Shehata et al., 2012; Donovan and Poronnik, 2013; Tang et al., 2014; Goo et al., 2017; Gerónimo-Olvera and Massieu, 2019; Yang, 2020). Nedd4, an E3 ubiquitin ligase, was found to ubiquitinate AMPA receptors and facilitate their endocytosis (Lin et al., 2011). In cultured rat hippocampal neurons, chemical induction of LTD with low-dose NMDA led to a concurrent increase of autophagosomes and degradation of AMPA receptor subunit, mGluR1 (Shehata et al., 2012).

Other studies have indicated that degradative pathways play an important and perhaps unexpected, role in LTP as well as LTD. Recycling endosomes, which break down membrane proteins in preparation for re-use by the cell, were found to be required for LTP in rat hippocampal slices, in a Rab11-dependent manner (Park et al., 2006). This study – which also found that recycling endosomes were exocytosed at higher rates following LTP stimulation – is perhaps explained by research which has shown that recycling endosomes function in non-canonical anterograde trafficking of AMPA receptor subunit GluA1 as part of the secretory system (Park et al., 2006; Tang, 2008; Bowen et al., 2017). Rab11 knockdown has also been separately confirmed to cause reduction in dendritic spine number as well as attenuated response to LTP in rat hippocampal slices (Siri et al., 2020). UPS is also implicated in LTP: inhibition of proteasomes caused a decrease in stimulus-evoked dendritic spine growth in hippocampal slice cells (Hamilton et al., 2012; Hamilton and Zito, 2013). Other work in rat hippocampal cultures has indicated that inhibiting lysosomes causes loss of excitatory synapses (Goo et al., 2017). Furthermore, hippocampal spine density increased after knockdown of E3 ubiquitin ligase HRD1 in rat neuronal cell cultures (Saldate et al., 2018). Though perhaps paradoxical on first glance, it is likely that UPS facilitates both LTD and LTP through complex ubiquitin signals (Mabb and Ehlers, 2018; Yun et al., 2018). Interestingly, the ubiquitination and degradation of Arc, the immediate early gene discussed in section “Protein Synthesis and Plasticity,” may inhibit AMPA receptor endocytosis; the net effect being that the UPS ceases LTD through Arc inhibition (Mabb and Ehlers, 2018). Though the exact mechanisms are still incompletely understood, the contribution of protein degradation systems to dendritic pruning and dynamic spine regulation are undeniable.

### Protein Degradation and Disease

Apart from dynamic changes of dendritic spine morphology, protein degradation systems are also involved in response to injury. Axonal Wallerian degeneration relies on autophagy, and although dendrites can be rapidly dismantled, it does not appear to occur through the same mechanisms as in axons, so autophagy may not be used in dendritic degeneration in the same way (Tao and Rolls, 2011; Yang et al., 2013). In mouse models with induced traumatic brain injuries, proteasomal activity decreased in the general area of injury, while autophagic markers increased; however, there is still some debate as to whether autophagy and UPS are neuroprotective or neurotoxic in cases of dendritic and axonal injury or ischemia (Yang et al., 2013; Feldmann et al., 2019; Gerónimo-Olvera and Massieu, 2019). Though it is still unclear how the two degradative subsystems interact, research indicates that they cooperate with one another in select situations (Yue et al., 2009; Feldmann et al., 2019). For comprehensive reviews on UPS-autophagy interactions see Lilienbaum (2013) and Kocaturk and Gozuacik (2018).

Like the other two arms of proteostasis, protein degradation is widely studied in neurodegenerative disease. E3 ubiquitin ligases, for example, have been linked to a wide range of neurodegenerative diseases; for a thorough review see George et al. (2018). Alterations to protein degradation have been implicated in a broad spectrum of neurodegenerative conditions including PD, HD, Multiple Sclerosis and ALS (Mitra et al., 2009; Koch et al., 2015; Albert et al., 2017; Mitsui et al., 2018). UPS and autophagy have become increasingly pursued areas of study in conjunction with neurodegenerative and protein aggregation diseases (George et al., 2018; Kabir et al., 2020; Kumar et al., 2020; Lambert-Smith et al., 2020; Lim et al., 2020; Papanikolopoulou and Skoulakis, 2020; Tundo et al., 2020).

The majority of research indicates that autophagic processes are neuroprotective, and disease-related dysfunction of autophagy contributes to or even causes neuronal degeneration (Yue et al., 2009; Yang et al., 2013; Deng et al., 2015; Koch et al., 2015; Albert et al., 2017; Beltran et al., 2019; Duan et al., 2019; Renaud et al., 2019). In ALS, for example, increased autophagy may help to clear mutant SOD1 protein and contribute to stalled degeneration of neurites in a human motor neuron cell culture model of familial ALS (Wu et al., 2019). Another instance of the neuroprotective function of autophagy is in PD models, with a recent study concluding that blockage of autophagic function causes increased levels of misfolded protein aggregation in human cell culture with induced human α-synuclein aggregates (Gerónimo-Olvera and Massieu, 2019). For recent reviews of the role of protein degradation systems in neurodegenerative disease see Kabir et al. (2020), Kumar et al. (2020), Lim et al. (2020).

The role of dendritic and spine morphology in the etiology of neuropsychiatric disorders is certainly not clear, but dysfunction of UPS and autophagy mechanisms have been linked to such disorders and are known to facilitate correct dendritic architecture. Nedd4, previously discussed for its role in AMPA receptor endocytosis, is an E3 ubiquitin ligases which facilitates proper formation of the dendritic arbor through ubiquitination of Rap2A (Donovan and Poronnik, 2013; Hamilton and Zito, 2013). Recently, single-nucleotide polymorphisms of the *Nedd4* gene have been linked to schizophrenia (Han et al., 2019). For an excellent summary of neurological disorders associated with dendritic spine abnormalities see (Chidambaram et al., 2019).

Many single-nucleotide polymorphisms associated with ASD symptoms are connected to failures in the autophagic and UPS systems (Bowling and Klann, 2014; Tang et al., 2014; Devitt et al., 2015; Louros and Osterweil, 2016). ASD symptomology is linked with dendritic overgrowth and increased density of spines (Hamilton and Zito, 2013; Louros and Osterweil, 2016). ASD has also been connected to instances when the E3 ubiquitin ligase UBE3A gene is duplicated or triplicated, which leads to increases in spine density (Yi et al., 2015). Dysregulations of autophagy are also associated with ASD, as knockdown of *Atg7* in microglia increased the number of somatosensory dendritic spines and caused ASD-like behavioral phenotypes to appear in mice (Kim et al., 2017). Conversely, deficiency of *Ube3A* is etiologically linked to AS with characteristic decreases in dendritic spine density thought to be caused by disinhibition of the PP2A phosphatase (Wang J. et al., 2019; Wang T. et al., 2019).

Though earlier discussed as a result of protein synthesis dysfunction, research implicates autophagy in Fragile X forms of ASD as well: hippocampal mouse neurons with no FMRP showed overactivation of mTORC1 – an autophagy inhibitor – causing the Fragile X model neurons to over-translate and under-degrade protein (Yan et al., 2018). These findings highlight both independent and interacting roles of proteostasis arms as checkpoints that must coordinately function in order to maintain a healthy cellular environment. It is an excellent example of the ways in which each arm of proteostasis is not only independently required, but work as a check on the other arms in order to maintain a healthy cellular environment.

## Discussion

Each arm of proteostasis – translation, maintenance, and degradation – is essential to maintaining the “status quo” of homeostasis in dendrites. However, these important molecular components should not be relegated to the category of “housekeeping” proteins. They participate in neuronal cell dynamics like dendritic spine remodeling and LTP/LTD as well as developmentally vital processes such as neurite outgrowth and cell-type specification. Ribosomes, chaperones, and the ubiquitin proteasome and autophagy systems are all heavily studied in relation to neurodegenerative disease. There is a newly revived interest in using endogenous proteostatic mechanisms to combat the neurobiological signs of disease; researchers are eager to overexpress or stimulate proteins that show decreased levels with age or disease. However, in many cases of neurodegeneration, the lowered expression of a protein may not be a cause of protein aggregation, but rather a symptom of the same molecular cascade which created the root problem. Protein aggregation itself, of course, can be a symptom of a problem higher in the chain, and one can see from reports of plaques and tangles in “normal” aging brains that aggregation itself is not the single cause of cognitive decline (Guillozet et al., 2003). The necessity of discovering the roles and interactions of the many players of protein quality control in healthy cells cannot be overstated. While molecular research of pathological states is incredibly important, and may lead us more quickly to discoveries that alleviate symptoms and slow progression, true understanding of the homeostatic mechanisms of these vital proteostatic processes may be the only route we have to a true molecular solution.

## Author Contributions

EL drafted the original manuscript. DC conceived the scope of the review and did the supervision. EL and DC critically analyzed, edited, and drafted final manuscript and did the funding acquisition. Both authors contributed to the article and approved the submitted version.

## Conflict of Interest

The authors declare that the research was conducted in the absence of any commercial or financial relationships that could be construed as a potential conflict of interest.
